# Structural insights into diversity and *n*-alkane biodegradation mechanisms of alkane hydroxylases

**DOI:** 10.3389/fmicb.2013.00058

**Published:** 2013-03-21

**Authors:** Yurui Ji, Guannan Mao, Yingying Wang, Mark Bartlam

**Affiliations:** ^1^Key Laboratory of Pollution Processes and Environmental Criteria (Ministry of Education), Tianjin Key Laboratory of Environmental Remediation and Pollution Control, College of Environmental Science and Engineering, Nankai UniversityTianjin, China; ^2^State Key Laboratory of Medicinal Chemical Biology, Nankai UniversityTianjin, China; ^3^College of Life Sciences, Nankai UniversityTianjin, China

**Keywords:** alkane hydroxylases, biodegradation mechanism, (an)aerobic, pMMO, sMMO, AlkB, cytochrome P450, LadA

## Abstract

Environmental microbes utilize four degradation pathways for the oxidation of *n*-alkanes. Although the enzymes degrading *n*-alkanes in different microbes may vary, enzymes functioning in the first step in the aerobic degradation of alkanes all belong to the alkane hydroxylases. Alkane hydroxylases are a class of enzymes that insert oxygen atoms derived from molecular oxygen into different sites of the alkane terminus (or termini) depending on the type of enzymes. In this review, we summarize the different types of alkane hydroxylases, their degrading steps, and compare typical enzymes from various classes with regard to their three-dimensional structures, in order to provide insights into how the enzymes mediate their different roles in the degradation of *n*-alkanes and what determines their different substrate ranges. Through the above analyzes, the degrading mechanisms of enzymes can be elucidated and molecular biological methods can be utilized to expand their catalytic roles in the petrochemical industry or in bioremediation of oil-contaminated environments.

Alkanes are major constituents of natural gas and petroleum. Many living organisms such as plants, green algae, bacteria, or animals can also produce alkanes. For example, methanogenic bacteria produce methane as a metabolic end product. Plants and animals can secrete alkanes as part of their surface waxes, which they use in order to prevent water loss (Cheesbrough and Kolattukudy, 1988). As the major components of petroleum and natural gas, alkanes play an important role in modern life. However, the inertness and viscosity of solid alkanes present a significant challenge for environmental scientists once they enter soil and water bodies via accidental oil spills and leakage. After considerable efforts to try to restore oil-contaminated soil and water, microbial degradation of these contaminants may present the best solution as a large number of microorganisms, which have various uptake mechanisms and enzyme systems, can grow by utilizing these contaminants as their sole carbon and energy source. In doing so, these microbes convert the inert alkanes into less inert substances that are easier to be oxidized and employed by other microorganisms. Accordingly, studying the enzyme systems employed by these alkane-degrading microorganisms has considerable importance for environmental and industrial applications. Understanding the mechanisms of alkane degradation by these enzyme systems may help in the control of oil pollution and in the modification of those energy-intensive industrial processes producing more valuable chemicals from the inert alkanes, such as converting methane to methanol.

In this review, we briefly summarize both the aerobic and anaerobic degradation pathways and degradation mechanisms of *n*-alkanes by microbes. We focus on the key enzymes involved in the initial activation step of aerobic degradation of *n*-alkanes, i.e., alkane hydroxylases, and discuss their structural features, structure–function relationship, and potential applications in industry.

## *n*-ALKANE DEGRADATION PATHWAY

Activation of alkanes by microbes can be carried out under both aerobic and anaerobic conditions with different enzyme systems. Under aerobic conditions, oxygen serves as the electron acceptor, while under anaerobic conditions, sulfate and nitrite accept electrons in order to complete the process.

### AEROBIC DEGRADATION PATHWAY

**FIGURE 1:**
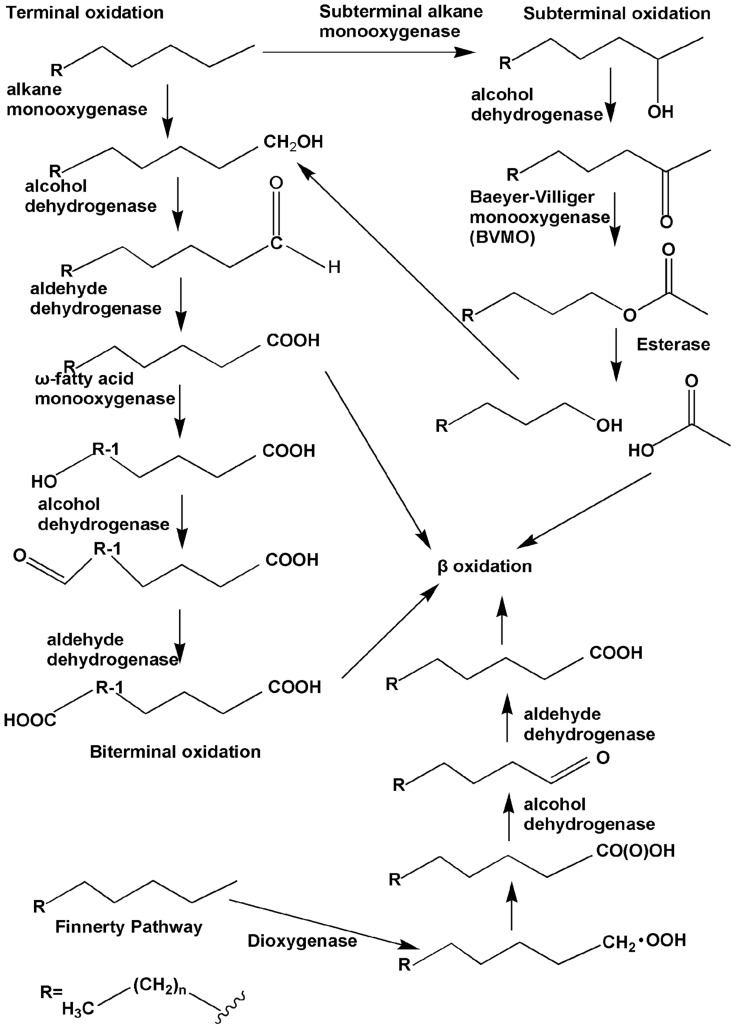
**The four types of aerobic degradation pathway (adapted from van Beilen et al., 2003).** Fatty acids produced via terminal oxidation can either enter β-oxidation or be oxygenated further by the ω-fatty acid monooxygenases to form dicarboxylic acid, i.e., biterminal oxidation. Subterminal oxidation leads to the secondary alcohol or methyl acetone, which can further be oxidated by the subsequent Baeyer–Villiger monooxygenase and esterase, generating an alcohol and a fatty acid. The Finnerty pathway is initiated by dioxygenases to form *n*-alkyl hydroperoxides, which are then in turn oxidized to peroxy acids, alkyl aldehydes, and fatty acids.

Under aerobic conditions, the alkane degradation reaction is initiated by oxygenases, which introduce oxygen atom(s) into alkane substrates. Four pathways for the initial attack on *n*-alkanes have been identified, and the reactions have been elucidated (**Figure 1**). First is the monoterminal (or terminal) oxidation pathway, which has been found in many bacteria such as *Geobacillus thermodenitrificans *NG80-2 (Li et al., 2008). In this pathway, the reaction proceeds as follows: alkanes are first attacked at their terminal methyl group to yield the corresponding primary alcohols, which are further oxidized by alcohol dehydrogenases and aldehyde dehydrogenases to fatty acids. The fatty acids then enter β-oxidation (Watkinson and Morgan, 1990). Second is biterminal oxidation, in which the termini of the *n*-alkane undergo oxidation to the corresponding fatty acid without rupturing of the carbon chain. In this pathway, the fatty acid produced in the monoterminal oxidation pathway undergoes ω-hydroxylation at the terminal methyl group (the ω position), yielding an ω-hydroxy fatty acid that is further converted to a dicarboxylic acid, which then also enters β-oxidation (Kester and Foster, 1963; Watkinson and Morgan, 1990; Coon, 2005). Subterminal oxidation has been recognized in *Pseudomonas aeruginosa *(Forney and Markovetz, 1970) and *Gordonia* sp. strain TY-5 (Kotani et al., 2003). This process takes place when alkanes are oxidized at the subterminal position to form a primary alcohol and a secondary alcohol or methyl acetone with the same chain length as the substrate (Forney and Markovetz, 1970). A recent study has also found subterminal oxidation of *n*-alkanes in the *Gordonia* sp. strain TY-5, degrading propane into a secondary alcohol. The secondary alcohol is converted to the corresponding ketone, and then oxidized by a Baeyer–Villiger monooxygenase to form an ester. The ester is hydroxylated by an esterase, generating an alcohol and a fatty acid (Kotani et al., 2007).

The three aforementioned pathways have been known for several decades and were verified through studies with bacteria from different genera. Enzymes participating in the first step of each of the three pathways, usually called alkane hydroxylases or alkane oxygenases, form the focus of this review.

Another long-chain *n*-alkane oxidation pathway is unique to *Acinetobacter* sp. strain HO1-N, as postulated by Finnerty (1988). In this pathway, it is proposed that *n*-alkanes are oxidized to form *n*-alkyl hydroperoxides and then peroxy acids, alkyl aldehydes, and finally fatty acids. The first step involves a dioxygenase, which has been reported to be common in *n*-alkane-using *Acinetobacter* spp. (Maeng et al., 1996). However, further studies are needed in order to elucidate this process in greater detail.

### ANAEROBIC DEGRADATION PATHWAY

**FIGURE 2:**
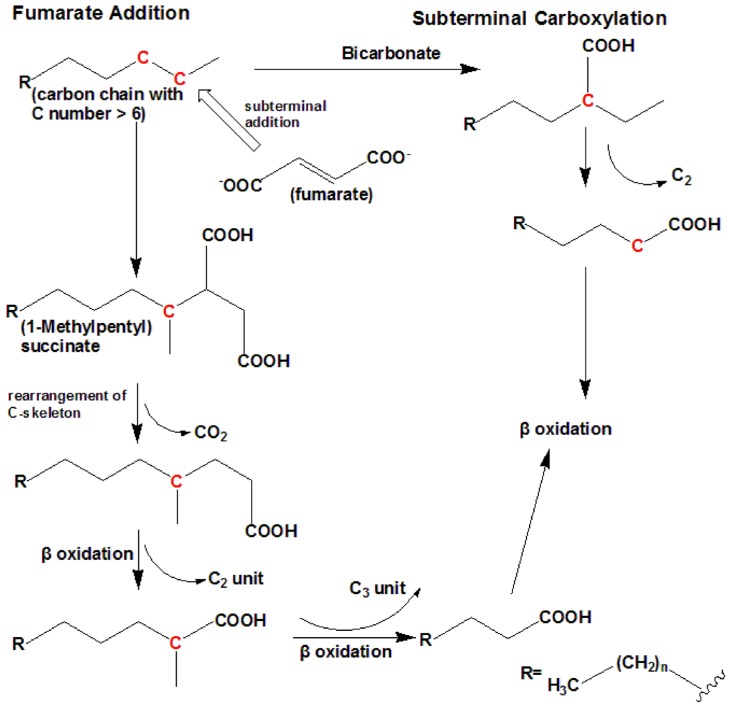
**The anaerobic degradation pathway (adapted from So et al., 2003; Cravo-Laureau et al., 2005).** It shows the two types of anaerobic degradation pathway that have currently been identified. To date, subterminal carboxylation has only been found in the sulfate-reducing bacterium strain Hxd3. For short-chain *n*-alkanes, such as propane, please refer to Kniemeyer et al. (2007).

Under anaerobic conditions, nitrate or sulfate is used as a terminal electron acceptor. To date, there are two known mechanisms of *n*-alkane anaerobic degradation (**Figure 2**). One is the fumarate addition pathway and the other is the carboxylation pathway. The anaerobic *n*-alkane degradation microorganisms that have been studied thoroughly are the sulfate-reducing bacterial strain AK-01 (So and Young, 1999), strain CV2803^T^ (Cravo-Laureau et al., 2005), strain Hxd3 (So et al., 2003), strain Pnd3 (Aeckersberg et al., 1998), and denitrifying bacterial strain HxN1 (Rabus et al., 2001). Anaerobic biodegradation of *n*-alkanes with bacterial enrichment culture has also been studied (Kropp et al., 2000; Callaghan et al., 2006, 2009). Strain Hxd3 is the first anaerobe shown to grow definitely on saturated hydrocarbons (Aeckersberg et al., 1991). Before the monooxygenase reaction was generally accepted as the initial step of alkane metabolism in aerobic microorganisms, it had been suggested that an oxygen-independent formation of a terminal double bond occurred as an alternative mechanism during aerobic growth on alkanes (Watkinson and Morgan, 1990). This mechanism also provided an explanation for the assumed anaerobic growth of certain bacteria on alkanes. It has previously been demonstrated that some microorganisms degrade *n*-alkanes via dehydrogenation and then addition of water to produce alcohols under anaerobic conditions (Parekh et al., 1977; Aeckersberg et al., 1991). Strain Hxd3 was later shown not to degrade alkanes anaerobically via a desaturation to the corresponding 1-alkenes (Aeckersberg et al., 1998). Instead, it transforms an alkane to a fatty acid via subterminal carboxylation at the C_3_ position of the alkane and elimination of the two adjacent terminal carbon atoms (So et al., 2003). Researchers observed that the initial attack of alkanes includes both carboxylation with inorganic bicarbonate and the removal of two carbon atoms from the alkane chain terminus, resulting in a fatty acid that is shorter by one carbon than the original alkane. In other words, following the degradation mechanism that strain Hxd3 employed, it can transform C-odd alkanes substrates to C-even fatty acids and vice versa (So et al., 2003). To date, it is the only isolated and identified strain that anaerobically degrades via the subterminal carboxylation pathway.

Fumarate addition proceeds via subterminal addition (at the C_2_ position) of the alkane to the double bond of fumarate, resulting in the formation of an alkylsuccinate. The alkylsuccinate is further degraded via carbon skeleton rearrangement and β-oxidation. The fumarate addition pathway has been found in both sulfate-reducing bacteria and denitrifying bacteria and a nitrate-reducing consortium (Kropp et al., 2000; Rabus et al., 2001; Cravo-Laureau et al., 2005; Davidova et al., 2005; Callaghan et al., 2006, 2009; Kniemeyer et al., 2007). For instance, the sulfate-reducing bacterium *Desulfatibacillum* *aliphaticivorans* strain CV2803^T^ oxidizes *n*-alkanes into fatty acids anaerobically via the addition of fumarate at C_2_ position, and unlike strain Hxd3, total cellular fatty acids of this strain had predominantly odd numbers of carbon atoms when the strain was grown on a C-odd alkane (pentadecane) and even numbers of carbon atoms when it was grown on a C-even alkane (hexadecane). The same is true for other strains employing the fumarate addition pathway (Cravo-Laureau et al., 2005). A more recent study on the *Desulfosarcina/Desulfococcus* cluster strain BuS5 degrading propane indicates a subterminal as well as a novel terminal alkane addition with fumarate, i.e., the fumarate adds to the primary carbon atom of propane (Kniemeyer et al., 2007).

It has been reported that different alkane degradation pathways could occur simultaneously within mixed sulfate-reducing consortia (Callaghan et al., 2006). To sum up, these findings underline that fumarate addition and carboxylation are important alkane anaerobic degradation mechanisms that may be widespread among phylogenetically and/or physiologically distinct microorganisms.

Anaerobic methane oxidation (AMO) has also recently been identified. Purified nickel-containing methyl-coenzyme M reductase (MCR) from *Methanothermobacter marburgensis* can convert methane into methyl-coenzyme M under equilibrium conditions; the apparent *V*_max_ (maximum rate) and *K*_m_ (Michaelis constant) are both consistent with the observed *in vivo* kinetics for the anaerobic oxidation of methane with sulfate (Scheller et al., 2010). In another recent study, AMO is also observed to be coupled with the reduction of nitrite to dinitrogen in an enrichment culture (Ettwig et al., 2010). This is a very interesting phenomenon as the anaerobic bacterium, *Methylomirabilis oxyfera*, essentially features a methane aerobic oxidation pathway with the oxygen derived from the conversion of two nitric oxide molecules. It remains to be seen how widespread this mechanism is among anaerobic bacteria, although such a process is predicted to offer certain ecological advantages for recalcitrant substrates including aromatic compounds, alkanes and alkenes under dynamic oxic/anoxic conditions. Microbial mats collected at cold methane seeps in the Black Sea have also been shown to oxidize methane anaerobically using sulfate as an electron acceptor (Mayr et al., 2008). These microbial mats predominantly consist of sulfate-reducing bacteria and archaea of the ANME-1 and ANME-2 type. Nevertheless, further studies are required to understand the mechanisms and the enzymes involved.

## DIVERSITY OF ALKANE HYDROXYLASES

Only four types of *n*-alkane aerobic degradation pathways have been identified to date, and the number of alkane hydroxylases that have been isolated, characterized, and analyzed by structural biology techniques remains limited. Studies have shown that they belong to different enzyme families. In the following section, we will classify the alkane hydroxylases into several groups according to their substrate range, degradation characteristics, and so on.

### METHANE MONOOXYGENASES AND PROPANE, BUTANE OXYGENASES

The first step in the catabolism of methane is catalyzed by methane monooxygenase (MMO) to form methanol. Methanol is then oxidized by methanol dehydrogenase to form formaldehyde, which is then converted to formate and carbon dioxide by formaldehyde and formate dehydrogenases to provide energy for the cell. It is reported that methanotrophs could also assimilate formaldehyde via the ribulose monophosphate pathway or serine pathway (Lieberman and Rosenzweig, 2004). Besides MMO-containing methanotrophic bacteria, only one other enzyme, ammonia monooxygenase, can activate the C–H bond in methane (Hyman et al., 1988). Due to the inert nature of methane (104 kcal mol^-^^1^ C–H bond), it has proven difficult for industries to convert it to methanol under ambient temperature and pressure (Lieberman and Rosenzweig, 2005).

There are two types of MMO: a membrane-bound, particulate MMO (pMMO) and a cytoplasmic, soluble MMO (sMMO). For most methanotrophs such as *Methylococcus capsulatus* (Bath), which contain both pMMO and sMMO, it is the concentration of copper ions in the medium that determines which MMO is expressed. Under low copper concentration conditions, the cells express only sMMO, while only pMMO is expressed when there is a high copper to biomass ratio (Stanley et al., 1983; Prior and Dalton, 1985; Murrell et al., 2000; Choi et al., 2003).

#### pMMO

Unlike sMMO, pMMO has a relatively narrow substrate specificity. It can only oxidize short chain *n*-alkanes (fewer than five carbon atoms). The oxidation of propane, *n*-butane and *n*-pentane preferentially occurs at the C_2_ position (Chan et al., 2004). Furthermore, it is reported that pMMO could also epoxidate alkenes at the double bond. Unlike the limited existence of sMMOs, pMMO is ubiquitous in methanotrophic bacteria. As a membrane-bound protein, difficulties in solubilizing and purifying active pMMO present challenges for understanding its structure, including concentration of metal ions and location of the active site. Furthermore, difficulties in obtaining enzymatically active, pure pMMO have led to conflicting results of *in vitro* biochemical characterization (Kitmitto et al., 2005). Two groups reported structures of pMMO from the methanotroph *Methylococcus capsulatus *(Bath) in 2005 by X-ray crystallography (Lieberman and Rosenzweig, 2005) or by electron microscopy and single-particle analysis (Kitmitto et al., 2005). The research findings from the two studies are consistent and provide structural information for an enzyme that play an important role in the transformation of methane to methanol, and which may have potential applications in tackling global warming given that methane is a type of greenhouse gas. The enzyme consists of a hydroxylase formed by three polypeptides with molecular masses of approximately 47 (α, pmoB), 26 (β, pmoA), and 23 kDa (γ, pmoC). The enzyme has also been reported to form a complex with an additional component called pMMO-R formed by two polypeptides with molecular masses of 63 and 8 kDa (Basu et al., 2003; Myronova et al., 2006), although this remains controversial. A further study showed that pMMO-R is methanol dehydrogenase, the subsequent enzyme in the methane oxidation pathway by methanotrophs (Myronova et al., 2006).

**FIGURE 3:**
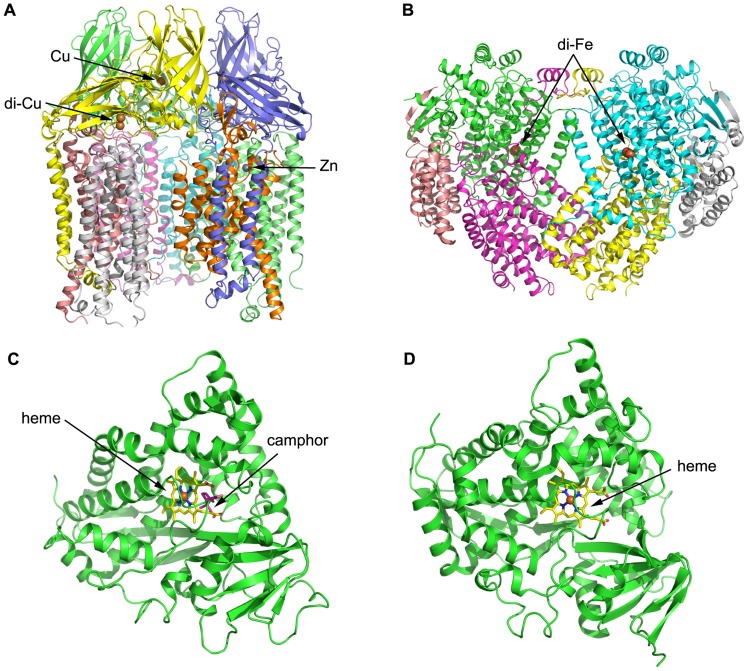
**Three-dimensional structures of alkane hydroxylase enzymes.** **(A)** The crystal structure of particulate methane monooxygenase (pMMO) from *Methylococcus capsulatus* (Lieberman and Rosenzweig, 2005). The structure is shown in cartoon representation and colored by chain; the Zn and Cu ions are shown as spheres. **(B)** The crystal structure of soluble methane monooxygenase (sMMO) from *Methylococcus capsulatus* (Rosenzweig and Lippard, 1994). The structure is shown in cartoon representation and colored by chain; the di-iron centers are shown as spheres. **(C)** The crystal structure of P450cam from *Pseudomonas putida* (Poulos et al., 1987). The structure is shown in cartoon representation, with the heme shown in stick representation and the iron shown as a sphere. Camphor is also shown in stick representation and colored magenta. **(D)** The crystal structure of P450 BM-3 from *Bacillus megaterium* (Ravichandran et al., 1993). The structure is shown in cartoon representation, with the heme shown in stick representation and the iron shown as a sphere.

Both pMMO structural studies confirmed that the hydroxylase adopts a cylindrical trimer with an α_3_β_3_γ_3_ polypeptide topology, with approximate dimensions of 105 Å in length and 90 Å in diameter (Lieberman and Rosenzweig, 2005; **Figure 3A**). A soluble region is composed of six β-barrel structures, two from each protomer, and extends approximately 45 Å from the membrane. pMMO is anchored into the membrane by 42 transmembrane (TM) helices, 14 from each protomer. An 11 Å hole in the center of the trimer in the soluble region is lined with glutamic acid, aspartic acid, and arginine residues, which serve to stabilize the trimer. This hole extends through the structure into the membrane where it is lined with hydrophobic residues and widens to approximately 22 Å at the opposing end to the soluble region.

Three metal centers were identified per protomer from the crystal structure: the first and second sites are located in pmoB, and the third site is located within the lipid bilayer. The first site contains a single metal ion assigned as copper, while the second metal site is a conserved dinuclear site that contains two copper ions, which was also found in subsequent pMMO structures from *Methylosinus trichosporium* OB3b (Hakemian et al., 2008) and *Methylocystis* species Strain M (Smith et al., 2011), respectively. The third metal center, modeled as a single zinc ion, is located within the lipid bilayer; it was proposed to be derived from the crystallization buffer but could be occupied by other metal ions *in vivo*. Structural analysis therefore identified a total of four metal ions per protomer, which differs significantly from other previous studies (Chan et al., 2004; Lieberman and Rosenzweig, 2005).

Activity experiments and mutagenesis have subsequently confirmed that the copper active site is located in the soluble domain of the pmoB subunit and is a dicopper center (Balasubramanian et al., 2010). The reactivity of a recombinant soluble fragment of the pmoB subunit (denoted as spmoB) and inactive spmoB, in which the dicopper center is disrupted, with oxidants were compared and an absorbance feature at 345 nm in spmoB was not produced in the inactive spmoB. Reaction of the 345 nm species with methane resulted in the disappearance of the spectroscopic feature, suggesting that this O_2_ intermediate should be mechanistically relevant. These observations support the idea that the dicopper center is the activation site and molecular oxygen binds at the dicopper center (Culpepper et al., 2012).

#### sMMO

Soluble MMO is a soluble, cytoplasmic monooxygenase that can oxidize a broad range of substrates including saturated alkanes, alkenes, aromatics, and chlorinated aromatics (McDonald et al., 2006). As sMMO is soluble and can oxidize many chemicals, it has drawn the attention and efforts of researchers and it is better understood than pMMO. sMMO systems isolated from *Methylococcus capsulatus* (Bath) and *Methylosinus trichosporium* OB3b have been studied extensively, and the first three-dimensional structure of one component of sMMO from *Methylococcus capsulatus* (Bath) was obtained in 1993 (Rosenzweig et al., 1993). sMMO is a complex enzyme system comprised of three protein components: MMOH, MMOR, and MMOB. MMOH is a 251-kDa heterodimeric hydroxylase with an α_2_β_2_γ_2_****topology, containing a carboxylate- and hydroxo-bridged dinuclear iron center. MMOR is a 38.5-kDa iron-sulfur flavoprotein that utilizes flavin adenine dinucleotide (FAD) and [2Fe-2S] cofactors to transfer electrons from NADH to the hydroxylase active site. MMOB is reported to regulate MMO reactivity (Merkx et al., 2001). The dioxygen activation reaction and substrate oxidation occurs at the di-iron centers in the α subunits of MMOH.

The structure of MMOH reveals a heart-shaped dimer, with the interface between the monomers forms a canyon that is approximately 80 Å × 40 Å × 20 Å in size (**Figure 3B**; Rosenzweig et al., 1993). The di-iron centers reside in the core of the α subunit and are coordinated by four glutamate and two histidine residues. Available MMOH_ox_ structures suggest that dioxygen-binding most likely occurs via replacement of the weakly coordinating bridging water molecule distal to the histidines. A hydrophobic substrate-binding pocket distal to the histidines houses the MMO active site and preferentially binds hydrophobic molecules, such as methane and dioxygen. Several possibilities have been raised for substrate ingress into and product egress from the active site. One possibility is that the substrate enters into the cavity via a gap between two helices that form part of the α subunit and constitute the di-iron centers. Another possibility is that the substrate enters the active site through one or more of the five hydrophobic cavities identified in the α subunit (Merkx et al., 2001).

Many spectroscopic techniques suggest that MMOB exerts its influences on MMOH by binding in the vicinity of the di-iron site and slightly altering its structure. The regulatory protein MMOB controls the substrate selectivity of MMO. MMOB binds to MMOH, which contains the active site, and appears to create a pore consistent with the size of methane in the active site. Mutagenesis of MMOB may therefore broaden the substrate range (Zhang et al., 2006).

MMOR contains one [2Fe-2S] cluster and one FAD cofactor, which both promote electron transfer from NADH to MMOH. The [2Fe-2S] cluster is located in the N-terminal portion of MMOR. The FAD cofactor is located in the C-terminal domain of MMOR, as is the NADH-binding region.

Soluble MMO utilizes a complex electron shuttle system with the function of NADH-oxidation and hydroxylation of methane played respectively by two proteins. Activation of dioxygen and the actual hydroxylation reaction both occur within the MMOH protein.

#### Other oxygenases for short-chain *n*-alkanes

Other gaseous alkanes are metabolized by strains expressing propane or butane monooxygenases (BMOs) that are related to pMMO or sMMO. A new isolate, *Gordonia* sp. strain TY-5, is capable of growth on propane and *n*-alkanes from C_13_ to C_22_ as the sole carbon source. A gene cluster designated prmABCD, which encodes the components of a putative dinuclear-iron-containing multicomponent monooxygenase, was cloned and sequenced. It was found that prmABCD disrupted mutants cannot grow on propane, suggesting that prmABCD gene products play an essential role in propane oxidation by the bacterium. Further studies show that it oxidizes propane via subterminal oxidation to 2-propanol via a monooxygenase (Kotani et al., 2003) and then further to acetone (Kotani et al., 2007). A gene cluster designated acmAB was cloned in which the acmA and acmB genes encode a Baeyer–Villiger monooxygenase and esterase, respectively. Further studies show that acmAB gene products play an important role in the metabolism of acetone derived from propane oxidation. The propane metabolism pathway in *Gordonia* sp. strain TY-5 started with the oxidization of propane to 2-propanol, which is further converted to acetone, followed by methyl acetate, which is finally oxidized to acetic acid and methanol (Kotani et al., 2007).

Butane monooxygenase from the Gram-negative β-proteobacterium *Thauera butanivorans*, previously called “*Pseudomonas butanovora*” (Dubbels et al., 2009), can oxidize alkanes C_2_–C_9_ (Halsey et al., 2006) and has received considerable attention from researchers. It is a three-component di-iron monooxygenase system that consists of an iron-containing hydroxylase (BMOH), a flavo-iron sulfur-containing NADH-oxidoreductase (BMOR), and a small regulatory component protein (BMOB). BMO has a strong regiospecificity of hydroxylation at the terminal carbon atom (Dubbels et al., 2007). Although BMO shares high homology with sMMO, researchers had not identified any oxidation of methane by BMO prior to the study by Halsey et al. (2006). Due to geometric constraints in the active site, product release is believed to be rate limiting, and accumulation of 23 μM methanol led to complete inhibition of methane oxidation in the *T. butanivorans* wild-type strain. Site-directed amino acid mutations were made in the α subunit of BMOH and a mutant form of BMOH found to partly abolish this restriction, resulting in a less methanol-sensitive enzyme that can tolerate a methanol concentration as high as 83 μM (Halsey et al., 2006). Propionate is a potent repressor of BMO expression: 10 μM propionate is able to reduce transcriptional activity of the BMO promoter. Propionate catabolism was inactive during growth of *Pseudomonas butanovora* on even-chain-length alkanes. However, propionate consumption was induced following growth on the odd-chain-length alkanes propane and pentane, resulting in a striking difference in the response to even- versus odd-chain-length alkanes (Doughty et al., 2006).

Recently, researchers have identified a novel membrane-associated monooxygenase (pBMO) from the Gram-positive bacterium* Nocardioides* sp. strain CF8 growing on butane. The arrangement of the genes encoding pBMO and the genes encoding pMMO from the methane-oxidizing bacteria are similar. Phylogenetic analysis suggests that pBMO represents a deeply branching third lineage of the bacterial family (Sayavedra-Soto et al., 2011), although further studies are needed to provide a comprehensive understanding of pBMO.

### THE AlkB FAMILY OF ALKANE HYDROXYLASES

The most widely characterized alkane degradation system is the Alk system of *Pseudomonas putida* GPo1 (commonly known as *Pseudomonas oleovorans *GPo1 = TF4-1L = ATCC 29347), which oxidizes C_5_–C_12_ *n*-alkanes to 1-alkanols (van Beilen et al., 1994). The 1-alkanol products are then sequentially converted to the corresponding aldehydes, carboxylic acids, and acyl-coenzymes A (CoAs), which then enter the β-oxidation pathway (Throne-Holst et al., 2007). The Alk system can also catalyze versatile reactions including the hydroxylation of linear and branched aliphatic, alicyclic, and alkylaromatic compounds, demethylation of branched methyl ethers, and epoxidation of terminal olefins (van Beilen et al., 2005). This enzyme system has also been reported to oxidize gaseous alkanes such as propane and *n*-butane (Johnson and Hyman, 2006).

The *Pseudomonas putida* GPo1 alkane hydroxylase system is composed of three components: alkane hydroxylase (AlkB), rubredoxin (AlkG), and rubredoxin reductase (AlkT; Smits et al., 2002). AlkB is a non-heme iron integral membrane protein that carries out the hydroxylation reaction (Kok et al., 1989; van Beilen et al., 1992). The NADH-dependent flavoprotein rubredoxin reductase transfers electrons from reduced nicotinamide to rubredoxin (Lode and Coon, 1971). Rubredoxin, a small red-colored iron-sulfur protein, transfers reducing equivalents to AlkB (McKenna and Coon, 1970; Smits et al., 2002).

The OCT plasmid of GPo1 encodes two rubredoxins, AlkF and AlkG. AlkG is unusual in that it is more than three times the size of other bacterial rubredoxins. It is composed of two rubredoxin domains connected by a 70-amino acid linker (Kok et al., 1989). Each domain binds a single iron atom, although the iron in the N-terminal domain is very loosely bound and is usually lost in the isolated protein (van Beilen et al., 1994). Rubredoxins cloned from microbes that grow on *n*-alkanes can be grouped into AlkG1- and AlkG2-type rubredoxins based on their amino acid sequences. All of the alkane-degrading strains contain AlkG2-type rubredoxins, whereas AlkG1-type rubredoxins are only present in a limited number of alkane-degrading strains. Two iron-binding CXXCG motifs are common to most alkane-degrading rubredoxins. Insertion of arginine downstream of the second CXXCG motif results in the failure of AlkG1 to transfer electrons to the alkane hydroxylase, thus providing a means of distinguishing AlkG1-type rubredoxins from the AlkG2-type rubredoxins.

Researchers have employed protein engineering to study the substrate specificity of AlkB and found that W55 (in the case of *Pseudomonas putida* AlkB) or W58 (in the case of *Alcanivorax borkumensis* AlkB1) plays a key role in determining the substrate range. Interestingly, mutation of this amino acid to a much less bulky amino acid enables AlkB in *Pseudomonas putida* to oxidize longer *n*-alkanes than the wild-type (van Beilen et al., 2005).

To date, there is no detailed structural information for AlkB, nor is there much biochemical data for AlkB with an extended substrate range. Nevertheless, a topology model of AlkB has provided some insight into its structure–function relationship. AlkB is predicted to contain six alpha-helical TM segments (van Beilen et al., 1992), which are thought to form a hydrophobic pocket. The N-terminus, two hydrophilic loops, and a large C-terminal domain are all located in the cytoplasm. Only three very short loops close to the amino acid positions 62, 112, and 251 are exposed to the periplasm (van Beilen et al., 1992). Mutagenesis experiments have verified that the eight histidines on the TM segment are very important for activity of AlkB, such as coordinating the Fe ions in the di-iron active site (Shanklin and Whittle, 2003). AlkB is proposed to belong to a larger family containing the non-heme integral membrane desaturases, epoxidases, acetylenases, conjugases, ketolases, decarbonylase, and methyl oxidases; all of the enzymes feature eight conserved histidine residues in a similar relative position with respect to the TM domains.

Researchers have also cloned novel genes encoding AlkB-rubredoxin fusion proteins from Gram-positive bacteria, such as *Dietzia* strain DQ12-45-1b, *Dietzia* sp. E1, *Prauserella rugosa* NRRL B-2295, and *Nocardioides* sp. strain CF8 (Hamamura et al., 2001; Smits et al., 2002; Bihari et al., 2011; Nie et al., 2011). The fusion protein encoded by *alkW1* from* Dietzia* strain DQ12-45-1b, consisting of an integral membrane alkane monooxygenase (AlkB) conserved domain and a rubredoxin conserved domain, can degrade C_8_–C_32_ *n*-alkanes and is experimentally verified to be favorable for the oxidation of long-chain alkanes. Two possibilities for the mechanism of the favorable oxidation have been proposed. The first involves steric effects on the binding between the substrate and enzyme, or between the enzyme and rubredoxin. The second is easier electron transport caused by a shorter distance between the two fused proteins. Phylogenetic analysis of the fused rubredoxins identified from different species clearly showed that all AlkB-fused rubredoxins constitute a novel third cluster of rubredoxins that are significantly distinct from either AlkG1-type or AlkG2-type rubredoxins. In addition, all AlkB-fused rubredoxins contain the conserved C(P/S)DCGVR motif in addition to the first CXXCG motifs (Nie et al., 2011).

### CYTOCHROME P450 ALKANE HYDROXYLASES

Cytochrome P450 enzymes are terminal monooxygenases that have been detected in nearly all domains of life, from prokaryotes such as *Pseudomonas putida* to eukaryotes, where yeasts can serve as an example. Their ubiquitous existence in nature determines their diverse substrate range, including fatty acids, steroids, prostaglandins, as well as many exogenous materials such as drugs, anesthetics, organic solvents, ethanol, alkylaryl hydrocarbon products, pesticides, and carcinogens (Bernhardt, 2006). Based on differences in the components of the P450 monooxygenase systems, they can be grouped into two classes. Class I P450s are represented by P450s isolated from mitochondria and bacteria. Electron transfer to these enzymes from NAD(P)H is mediated by an FAD-containing reductase (which accepts electrons from NAD(P)H) and an iron-sulfur protein (which shuttles electrons from the reductase to the substrate-bound P450; Huang and Kimura, 1973). The class II P450s consist of microsomal drug-metabolizing forms that receive electrons from NAD(P)H via an FAD- and flavin mononucleotide (FMN)-containing cytochrome P450 reductase (van Beilen and Funhoff, 2005). P450s from bacteria are mostly soluble while P450s from yeast and mammals are usually membranous, which makes their further study more challenging. At the time of writing, more than 4,000 P450 enzymes are known (van Beilen and Funhoff, 2005). However, with regard to P450s degrading *n*-alkanes, the number is rather small.

To date, the P450s that have received the most attention and been extensively studied are the P450cam system from *Pseudomonas putida* ATCCl7453, which require putidaredoxin and putidaredoxin reductase to transfer electrons from NADH to P450cam for the oxidation of camphor to 5-exo-hydroxycamphor (**Figure 3C**; Trudgill et al., 1966; Poulos et al., 1987), and P450 BM-3 from *Bacillus megaterium* 14581 that hydroxylates medium-chain (from C_12_ to C_18_) saturated fatty acids (**Figure 3D**; Narhi and Fulco, 1986; Ravichandran et al., 1993). P450 BM-3 is the most active of all P450 enzymes, and this has been suggested to be due to the fusion of a hydroxylase domain and a reductase domain into a single polypeptide chain, which is different to other P450 enzymes (Narhi and Fulco, 1986). Although these two enzymes have been studied intensively, none of their substrates are *n*-alkanes, which are the most inert molecules. Researchers have thus employed molecular biology and other related methods in combination with structural information to modify the substrate-binding pockets and active sites of the enzymes, in order to make them more suitable for binding to and degrading smaller *n*-alkane molecules. For instance, P450cam was engineered into an alkane hydroxylase with amino acid residues in the active site replaced by residues with bulkier and more hydrophobic side chains. The resulting mutant (F87W/Y96F/T101L/V247L) had a comparable catalytic turnover rate for *n*-butane oxidization to that of the wild-type and exhibited the highest propane oxidation rate of the P450cam enzymes studied (Bell et al., 2003). Rational evolution of P450 BM-3 produced a triple mutant (Phe87Val, Leu188Gln, Ala74Gly) that is capable of oxidation of *n*-octane at a similar rate as *n*-dodecanoic acid (Appel et al., 2001). Directed evolution of P450 BM-3 produced mutant 139-3 that can degrade *n*-alkanes (from C_3_ to C_8_) at the subterminal position, which resembles the native enzyme’s regioselectivity on fatty acids (Glieder et al., 2002). CYP102A3 from *B. subtilis* hydroxylates medium-chain fatty acids in subterminal positions, as does P450 BM-3 (CYP102A1) from *B. megaterium *(Whitehouse et al., 2010). Two CYP102A3 mutants can oxidize octane with ratios of 43% (S189Q) and 49% (F88V/S189Q), respectively, which shows that F88 and S189 are important in determining the substrate spectrum of CYP102A3 (Lentz et al., 2004).

CYP153 enzymes are class I P450 proteins requiring the presence of an electron-delivering protein system (ferredoxin and ferredoxin reductase protein). Cytochrome P450 enzymes from the CYP153 family are the first soluble P450 enzymes that specifically display hydroxylating activity toward the terminal position of alkanes (Scheps et al., 2011). CYP153A6 from *Mycobacterium* sp. HXN-1500 is the first soluble P450 that hydroxylates unreactive aliphatic alkanes, mainly medium-chain-length alkanes (from C_6_ to C_11_), with high regioselectivity on terminal positions to 1-alkanols. Longer alkanes bind more strongly than shorter alkanes, while the introduction of sterically hindering groups reduces the affinity. This suggests that the substrate-binding pocket is shaped such that linear alkanes are preferred (Funhoff et al., 2006). CYP153 is a cytochrome P450 from *Acinetobacter* sp. EB104, which constitutes a new P450 family; it catalyzes the hydroxylation of unsubstituted *n*-alkanes (Maier et al., 2001). CYP153C1 from the oligotrophic bacterium *Novosphingobium aromaticivorans* DSM 12444 can bind linear alkanes such as heptane, octane, and nonane (Zhou et al., 2011).

Several species of yeast belonging to the genus* Candida* excrete α,ω-diacids as a by-product when grown on *n*-alkanes or fatty acids as the carbon source. One such yeast species, *Candida tropicalis* ATCC 20336, has been studied in detail in regard to the CYP52 family, which is important for the conversion of *n*-alkanes and fatty acids to α,ω-dicarboxylic acids. For alkanes, the first reaction occurs in the ω-oxidation pathway with the formation of the corresponding alcohol. A cytochrome P450 hydroxylase complex, which composes of a cytochrome P450 monooxygenase and the accompanying NADPH cytochrome P450 reductase, is responsible for the first and rate-limiting step of ω-oxidation of *n*-alkanes and fatty acids (van Beilen and Funhoff, 2005).

### LONG-CHAIN ALKANE MONOOXYGENASE (LadA)

**FIGURE 4:**
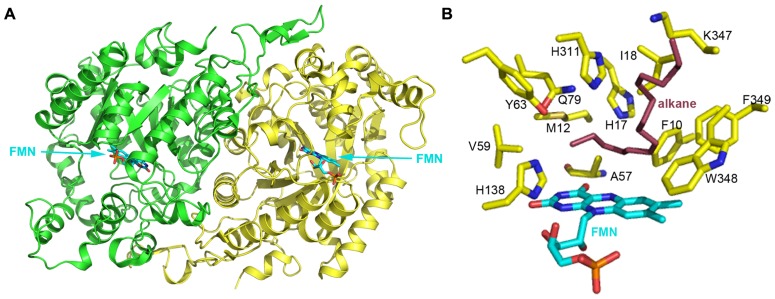
**(A)** The crystal structure of long-chain alkane monooxygenase (LadA) from *Geobacillus thermodenitrificans* NG80-2 (Li et al., 2008). The structure is shown in cartoon representation and colored by chain, with the FMN cofactor shown in stick representation and colored cyan. **(B)** The putative active pocket lined by several residues above the FMN *Si*-face. H138 possibly acts as a proton donor in the reaction between reduced flavin and oxygen that leads to the formation of the C4a-hydroperoxyflavin intermediate. A hydrophobic cavity formed by M12, A57, V59 and the 4′ OH group of the FMN ribityl chain and the terminal carbon of the alkane chain act to protect the C4a-hydroperoxyflavin from solvent. H17, Y63, Q79, and H311 are believed to be involved in substrate activation and electron transfer during hydroxylation. F10, I18, K347, W348, and F349 should be involved in binding the alkane substrate. LadA amino acids are shown as yellow sticks and labeled; the FMN coenzyme is shown in cyan stick representation. A docked alkane substrate is shown in red stick representation and labeled.

Several bacterial strains can assimilate *n*-alkanes with carbon chain length longer than C_20_. However, enzymes involved in these degradation processes usually do not belong to the three groups mentioned above. To date, few long-chain alkane hydroxylases have been cloned and characterized. Three-dimensional structures of long-chain alkane hydroxylases remained unclear until the 2.7 Å apoenzyme and 1.9 Å holoenzyme structures of LadA, a long-chain alkane monooxygenase, were reported in 2008 (**Figure 4A**; Li et al., 2008). LadA, isolated from the thermophilic bacillus *Geobacillus thermodenitrificans *NG80-2, utilizes a terminal oxidation pathway for the conversion of long-chain *n*-alkanes (from C_15_ to at least C_36_) to corresponding primary alcohols. The terminal oxidation pathway from *Geobacillus thermodenitrificans *NG80-2 has been well characterized and consists of three components: LadA, which is the key initiating enzyme; two alcohol dehydrogenases (ADH1 and ADH2) for the conversion of alkyl alcohols to alkyl aldehydes (Ji et al., 2013); and an aldehyde dehydrogenase (ALDH) for the conversion of alkyl aldehydes to fatty acids (Feng et al., 2007).

LadA was revealed to belong to the SsuD subfamily of the bacterial luciferase family via a surprising structural relationship (Li et al., 2008). The structure of LadA contains a triosephosphate isomerase (TIM) barrel fold that differs from the prototypical TIM barrel structure due to five extended insertion regions (IS1-5) and an extension at the C-terminus of the polypeptide chain (Li et al., 2008). A pocket at the C-terminal entrance of the TIM barrel is sufficiently large enough to accommodate a FMN, O_2_, and part of the terminal of a long-chain *n*-alkane. LadA was thus confirmed to be a flavoprotein monooxygenase that utilizes dioxygen to insert an oxygen atom into the substrate. The flavin ring of FMN lies in the barrel with its plane almost parallel to the staves of the barrel and its *Si*-face exposed to solvent (**Figure 4B**). The ribityl side chain and phosphate moieties insert between strands β4 and β5 of LadA in an elongated manner. A cavity above the *Si*-face of FMN is lined by the residues F10, M12, H17, A57, V59, Y63, Q79, H138, H311, W348, and F349, with polar residues concentrated on the left sphere and hydrophobic residues on the right sphere (**Figure 4B**).

In the absence of a LadA:FMN:alkane ternary complex, *in silico* docking of C_15_–C_22_ alkanes was used to provide insights into substrate binding and the terminal hydroxylation of long-chain aliphatic alkanes. All substrates were identically coordinated with the terminal carbon located above the FMN *Si*-face, located close to the C4a atom, and interposing the cavity mentioned above. The carbon chain adopts a sinuous conformation along the surface of the insert region IS4, with its terminal lying parallel to the plane of flavin *Si*-face. The substrates bind to the protein via hydrophobic interactions between the majority of the alkane chain and a cluster of hydrophobic residues in LadA, including F10, I18, K347, W348, and F349 (**Figure 4B**). Li et al. (2008) speculated that the substrate-binding mode requires substrate specificity and thus determines the range of the alkane carbon chain length for catalysis. It was predicted that alkane chains of C_14_ and lower would be of insufficient length for the terminal carbon to reach the active site while the opposing terminal part was anchored to the protein.

As the carbon–hydrogen bond is inert in aliphatic alkanes, the activation of the substrate is rationally required before it reacts with the C4a-hydroperoxyflavin intermediate. Four polar residues, H17, Y63, Q79, and H311, are located above the terminal carbon of the alkane (**Figure 4B**). Enzyme inactivation activity assays performed on single point mutants indicate that mutation of each of these four residues completely abolishes the catalytic activity of LadA. Thus, they are likely to play crucial roles in the catalytic reaction, and it was suggested that their polar side chains may be involved in substrate activation and electron transfer. Mutation of a fifth residue, C_14_, was also shown to abolish LadA catalytic activity, but this was achieved by disrupting the homodimer interface, indicating that dimerization is also important for catalytic activity. Although the process of activation and the precise catalytic mechanism could not be elucidated from the reported LadA structure, the structural analysis of LadA has provided a rational basis for further biochemical studies.

A recent study by Dong et al. (2012) reported the use of random- and site-directed mutagenesis to enhance the activity of LadA. Three mutants, A102D, L320V, and F146C/N376I from random-mutagenesis, together with six more mutants, A102E, L320A, F146Q/N376I, F146E/N376I, F146R/N376I, and F146N/N376I from site-directed mutagenesis, were obtained and the hydroxylation activity of purified mutants on hexadecane was found to be between 2- and 3.4-fold higher than that of the wild-type LadA, with the activity of F146N/N376I being the highest. In the same study, *Pseudomonas fluorescens* KOB2δ1 strains expressing the LadA mutants were found to grow more rapidly with hexadecane than the strain expressing wild-type LadA, confirming the enhanced activity of LadA mutants *in vivo* (Dong et al., 2012). Both the wild-type LadA and mutants were active at temperatures ranging from 40 to 90°C and at pH values from 6.0 to 8.8. This study thus confirms the suitability of LadA for industrial utilization.

Another noteworthy phenomenon is that although LadA belongs to the flavoproteins, the activity of the enzyme appears to be independent of flavin reductase, as indicated by *in vitro* activity assays performed with and without the presence of a reductase. Flavoprotein monooxygenases catalyze an overall reaction involving three general chemical processes: (a) reduction of the cofactor flavin by NAD(P)H; (b) reaction of the reduced flavin with O_2_ to provide a C4a-flavin(hydro)peroxide, which is the oxygenating reagent; and (c) binding, orienting, and activating the substrate for oxygenation by C4a-(hydro)peroxyflavin. A new group of flavoprotein monooxygenases has recently been identified that consist of two components: a NAD(P)H-dependent flavin reductase for reduction of flavin, and a monooxygenase that uses reduced flavin as a substrate for the oxidation reaction. The luciferase systems, to which LadA is related, are the first and the most extensively studied two-component flavin-dependent oxygenases. It was previously proposed that electrons may be directly transferred from NAD(P)H to the flavin during hydroxylation in some flavoproteins, including the well-studied PHBH (p-hydroxybenzoate hydroxylase; Moonen et al., 2002), and LadA. However, this hypothesis needs to be further confirmed (Dong et al., 2012).

### OTHER LONG-CHAIN *n*-ALKANE HYDROXYLASES

Other studies focused on long-chain alkane degradation have remained mainly at the level of genetic studies, with little or no further research on the enzymes involved. A novel dioxygenase isolated from *Acinetobacter* sp. strain M-1 utilizes *n*-alkanes ranging in length from C_10_ to C_30_ as its sole carbon and energy source with the presence of FAD and Cu^2^^+^ for its activity via the Finnerty pathway (Maeng et al., 1996). *Acinetobacter haemolyticus* strain AR-46, which is evolutionally distant from the known hydrocarbon-degrading *Acinetobacter* spp., is reported to be able to utilize long-chain *n*-alkanes ranging from C_16_ to C_35_ through the monoterminal oxidation pathway (Bihari et al., 2007). Based on these results, it is evident that an alkM encoded non-heme iron integral membrane alkane hydroxylase is the first key enzyme in the monoterminal oxidation pathway.

*Acinetobacter* sp. strain DSM 17874 can utilize C_10_–C_40_ *n*-alkanes as its sole carbon source (Throne-Holst et al., 2007). *almaA* was identified as the gene encoding a putative flavin-binding monooxygenase which enables the strain to utilize long-chain alkanes (>C_32_). An interesting phenomenon whereby an *almA*-deficient mutant was still able to grow with C_24_ and shorter alkanes as its sole carbon source, indicated that the same strain uses another enzyme system for degradation of shorter alkanes (Throne-Holst et al., 2007). By using highly degenerate primers, Liu et al. (2011) amplified the *almA *gene, with a *n*-alkane substrate range from C_22_ to C_36_, from the marine alkane degrader *Alcanivorax dieselolei* B-5. This substrate range complements those of *alkB* and *p450* genes that also exist in the strain. Studies using the same methods indicate that *almA* is more likely to be found in marine hydrocarbon-degrading bacteria, which implies that the *almA* gene is very important for the degradation of long-chain alkanes in the ocean (Wang and Shao, 2012).

## OUTLOOK

Alkane hydroxylases are widespread in petroleum-degrading bacteria and a number of them are quite efficient in oxidizing substrates. Therefore, there is considerable interest in employing alkane hydroxylases for industrial applications. Thermophilic long-chain *n*-alkane-degrading bacterial strains are of particular interest for their biotechnological applications. There are several advantages of using thermophilic microorganisms for bioremediation of hydrocarbons over mesophilic organisms. Generally speaking, elevated temperature can increase the solubility of hydrophobic pollutants, decrease their viscosity, enhance their diffusion, and transfer long-chain *n*-alkanes from the solid phase to liquid phase (Feitkenhauer et al., 2003). Most alkane hydroxylases are relatively complex and difficult to use *in vitro* as they are composed of multiple components, such as the P450 system and the AlkB system, leading to low electron transfer rates. They also usually require the presence of cofactors, which are sensitive to inactivation by activated oxygen species and sensitive to product inhibition. Furthermore, the substrates and the products of these enzymes tend to be quite hydrophobic and toxic to the host cell. Enzymes such as the long-chain monooxygenase LadA could be a favorable candidate in industrial oxygenation reactions as it meets most of the demands of an ideal enzyme used in industry (van Beilen and Funhoff, 2007): it is cofactor-independent, less sensitive to high temperature, and abundant quantities of the enzyme can be produced through heterologous expression in *E. coli*. Although there are problems that hinder their application in industry, protein engineering, site-directed mutagenesis and random mutagenesis, together with structural information for such enzymes, can help to overcome any problems and aid the development of alkane hydroxylase enzymes into efficient, highly selective catalysts.

## Conflict of Interest Statement

The authors declare that the research was conducted in the absence of any commercial or financial relationships that could be construed as a potential conflict of interest.
